# Book Reviews

**Published:** 1872-07-01

**Authors:** 


### Book Reviews.

We have received, through the bookstore of Jansen, McClurg & Co., of this city, the following valuable medical works:

Lectures on the Principles and Practice of Physic. Delivered at King's College, London, by Thomas Watson, Bart., M.D., F. R.S., Physician Ordinary to the Queen, Fellow and late President of the Royal College of Physicians of London, etc., etc., etc. Two volumes. From the fifth revised and enlarged English edition. Edited with additions and illustrations, by Mary Hartshorne, A.M., M.D., Professor of Hygiene in the University of Pennsylvania, etc., etc. Philadelphia: Henry C. Lea. 1872.

This is one of the most readable and interesting works on the practice of medicine, in our language; but its merits are so well known to our readers, that further comments are unnecessary.

---

A System of Surgery; Pathological, Diagnostic, Therapeutic, and Operative. By Samuel D. Gross, M.D., LL.D., D.C.L., Oxon. Professor of Surgery in the Jefferson Medical College of Philadelphia, etc., etc. Illustrated by upwards of 1400 engravings. Fifth edition, greatly enlarged and thoroughly revised. In two volumes. Philadelphia: Henry C. Lea. 1872.

This, also, is a new edition of a work well known to the profession as a full and complete treatise, embracing the whole field of surgery. The present edition has been thoroughly revised by the author, and is truly a monument to his industry and practical experience. The publishers have also done their work well in the mechanical execution of these two ponderous volumes. We commend the work unreservedly to the patronage of the profession.

---

The following have been received through the bookstore of W. B. Keen, Cooke & Co., of this city:

The Year-Book of Therapeutics, Pharmacy, and Allied Sciences. Edited by Horatio C. Wood, Jr., M.D., Professor of Medical Botany, University of Pennsylvania, Physician and Lecturer on Clinical Medicine to the Philadelphia Hospital, etc., etc. New York: William Wood & Company. 1872.

This is a neatly published volume of 360 pages. Its contents are divided into five parts, namely: Therapeutics; Materia Medica; Toxicology; Prescriptions and Formulas; General Recipes. Under these several heads the editor has arranged abstracts from the current periodical literature of the year, sufficient to give a fair indication of the progress made in these important departments of medical science. It will be found a very convenient and valuable book of reference.

---

Half-hour Recreations in Popular Science. Edited by Dana Estis. No. 4. Spectrum Analysis Discoveries. Showing its Application in Microscopical Research, and to Discoveries of the Physical Constitution and Movements of the Heavenly Bodies. From the works of Schellen, Young, Roscoe, Lackyer, Huggins, and others. Boston: Lee & Shepard. New York: Lee, Shepard & Dillingham. Price, 25 cents.

This is the fourth number of a monthly series, devoted to popular science. It contains 48 neatly printed pages, with several well executed illustrations. The subjects discussed are correctly indicated by the title page just quoted. The whole series will be found highly entertaining and instructive.

---

Bromide of Potassium.—A lad of twenty years of age, after taking half-drachm doses of bromide of potassium twice daily for a fortnight, complainted of loss of memory, and on inquiry Dr. J. Haddon, (*British Medical Journal*) found that the patient called things by their wrong names, and very often forgot the name he desired to pronounce.

